# Indirect effects of habitat disturbance on invasion: nutritious litter from a grazing resistant plant favors alien over native Collembola

**DOI:** 10.1002/ece3.1483

**Published:** 2015-07-25

**Authors:** Hans Petter Leinaas, Jan Bengtsson, Charlene Janion-Scheepers, Steven L Chown

**Affiliations:** 1 Department of Bioscience, University of OsloPO Box 1066, 0136, Oslo, Norway; 2 Department of Ecology, Swedish University of Agricultural Sciences (SLU)Box 7044, 750 07, Uppsala, Sweden; 3 School of Biological Sciences, Monash UniversityMelbourne, Victoria, 3800, Australia; 4 Centre for Invasion Biology, Department of Botany and Zoology, Stellenbosch UniversityPrivate Bag X1, 7602, Matieland, South Africa

**Keywords:** Facilitation, nonadditive effects, renosterveld, resource patchiness, South Africa

## Abstract

Biological invasions are major threats to biodiversity, with impacts that may be compounded by other forms of environmental change. Observations of high density of the invasive springtail (Collembola), *Hypogastrura manubrialis* in heavily grazed renosterveld vegetation in the Western Cape, South Africa, raised the question of whether the invasion was favored by changes in plant litter quality associated with habitat disturbance in this vegetation type. To examine the likely mechanisms underlying the high abundance of *H. manubrialis*, cages with three types of naturally occurring litter with different nutrient content were placed out in the area and collected after different periods of time. *Hypogastrura manubrialis* was mainly found in the nutrient-rich litter of the yellowbush (*Galenia africana*), which responds positively to disturbance in the form of overgrazing. This suggests that invasion may have been facilitated by a positive interaction with this grazing resistant plant. By contrast, indigenous Collembola were least abundant in yellowbush litter. Negative correlations between high abundance of *H. manubrialis* and the abundance and diversity of other species suggest that competitive interactions might underlie low abundance of these other species at the patch level. Group behavior enables *H. manubrialis* to utilize efficiently this ephemeral, high quality resource, and might improve its competitive ability. The results suggest that interactions among environmental change drivers may lead to unforeseen invasion effects. *H. manubrialis* is not likely to be very successful in un-grazed renosterveld, but in combination with grazing, favoring the nutrient-rich yellowbush, it may become highly invasive. Field manipulations are required to fully verify these conclusions.

## Introduction

Biological invasions are a major threat to biodiversity as a consequence of a range of substantial impacts (e.g., Mack et al. 2000; Pyšek et al. 2012). Other forms of environmental change may compound these threats and complicate predictions of impacts of invasive species on native communities and ecosystems (e.g., Didham et al. 2007). Nonetheless, the effects of different environmental change drivers are often examined independently, with the consequence that the outcomes of interactions among co-occurring drivers are not well understood (Brook et al. 2008; Walther et al. 2009; Chown et al. 2010). Such interactions may result in pronounced nonadditive effects (Didham et al. 2007; Crain et al. 2008; Darling and Côté 2008), often with unexpected consequences (Brook et al. 2008; Winder et al. 2011).

The relationship between habitat disturbance and species invasion has been widely explored (e.g., Marvier et al. 2004; Ewers and Didham 2006; Didham et al. 2007; Foxcroft et al. 2011). Success of invasive species is often facilitated by habitat disturbance, either as a direct response to disturbance or indirectly by responses of one species facilitating the invasion of another (e.g., Simberloff and Von Holle 1999; Richardson et al. 2000; Maron and Vilà 2001). In comparison, the interactive effects of habitat disturbance and invasion on indigenous species and ecosystems may be more complex, and causality not always straightforward to discern (MacDougall and Turkington 2005; Didham et al. 2005; but see Light and Marchetti 2007). Disturbance may affect indigenous species directly, and indirectly via its impact on the invasive species, and not necessarily in the same way (e.g., Davies et al. 2005; Fridley et al. 2007; Melbourne et al. 2007).

Impacts of invasions and their interactions with other drivers of environmental change have mainly focused on plants and on aquatic systems (see discussion in Herben et al. 2004; Chown et al. 2007; Pyšek et al. 2008). Less attention has been given to identifying the interactive effects of habitat modification and invasion on terrestrial arthropods, especially where habitat modification facilitates invasion by arthropods (see e.g., King and Tschinkel 2008). That is, several studies have dealt with effects of landscape modification on invertebrate invasions or of plant invasions on invertebrate diversity (e.g., Steenkamp and Chown 1996; Kappes et al. 2007; Samways & Sharratt 2010; Simao et al. 2010; Wolkovich 2010), but the interactions are less frequently investigated. In the absence of a broader set of experimental investigations, generality regarding the outcomes of interactive effects of habitat modification and invasion will remain elusive, especially as it appears that responses by the litter-dwelling and above-ground components of the fauna may be quite different (Wolkovich 2010). Improving general understanding of impacts has been identified as an important goal of invasion biology (e.g., Hulme et al. 2013; Ricciardi et al. 2013).

In this study, we therefore investigate the way in which habitat disturbance might affect the success of invasive relative to indigenous Collembola (springtail) species by changing the amount of high quality litter. In an area of renosterveld vegetation of the Western Cape province of South Africa, we observed mass occurrence of the European Collembola species *Hypogastrura manubrialis* (Tullberg), in particular in patches dominated by the yellowbush, *Galenia africana* L. This is a native plant species, that is, rare in undisturbed renosterveld, but favored by disturbances such as overgrazing (Allsopp 1999). In the same area, we documented variation in litter quality between different dominant plant species (Bengtsson et al. 2011). The Collembola are linked by diet to decomposing plant litter, and as *H. manubrialis* typically is associated with very nutrient-rich organic soils (e.g., Fjellberg 1998), and is exceptionally rare or absent from undisturbed, nutrient poor sites across the Western Cape (Janion 2012; Liu et al. 2012; Janion-Scheepers et al. 2015), we hypothesized that the invasive success of this species is favored by the rich yellowbush litter, which is promoted by habitat disturbance. Specifically, we examined the effect of experimental supply of yellowbush litter and two other litter types typical of undisturbed renosterveld vegetation on *H. manubrialis*, and on the indigenous species in the area. Interactions between the responses of these two Collembola groups were also investigated.

## Material and Methods

### Study site

The study area falls in the Fynbos Biome of the Western Cape Province of South Africa. The richer parts of this biome were originally covered by renosterveld, a shrub vegetation type dominated by renosterbos (*Dicerothamnus rhinocerotis* (L. f.) Koekemoer), of which only small remnants or habitat islands of native vegetation remain in a matrix of agricultural land (see Mucina and Rutherford 2006). The originally sparsely occurring native yellowbush (*G. africana*) has been strongly favored by overgrazing in many areas (Van der Lugt et al. 1992; Allsopp 1999). It is poisonous to livestock and has a much more nutrient-rich litter than the more typical plant species of the vegetation type, with, for example, twice as high concentrations of N and P and decomposing three times faster than the renosterbos litter (Bengtsson et al. 2011). Faster decomposing nutrient-rich litter leads to higher abundance of bacteria and fungi on which the Collembola feed (Hopkin 1997). Thus, the high litter quality is expected to affect the Collembola indirectly via the effect on the decomposing microflora. We selected two renosterveld sites near Piketberg, about 200 km North of Cape Town, on the farms Rhenosterhoek (32°32′S, 18°49′E, 1 ha site), and Meerlandsvlei (32°34′S, 18°53′E, 0.5 ha site). They were 7 km apart, substantially affected by livestock grazing, with both renosterbos and yellowbush being common (see Bengtsson et al. 2011 for a more detailed description).

### Study species

Collembola are typically soil-dwelling invertebrates that are abundant and play key roles in soil ecosystems (Hopkin 1997). Although the South African Collembola fauna is not well known, we have recently improved knowledge for the study area and its surroundings (e.g., Janion et al. 2011; Janion-Scheepers et al. 2015). In addition to the invasive species *H. manubrialis*, we have identified 15 other distinct morphospecies in the study area, with names available mostly to the genus or family levels (Table S1). *Hypogastrura manubrialis* was recorded in the Western Cape at least by the 1930s (Janion-Scheepers et al. 2015) and is of European origin, where it is typically found in rich organic soils such as compost (Fjellberg 1998) and has repeatedly been reported as a pest in mushroom farms (Ripper 1930; Simon 1975; Greenslade and Clift 2004).

### Plant species and experimental design

We used three common renosterveld plant species differing in nutrient content for the experiment. In addition to the yellowbush (*G. africana*), we selected the other dominant bush in the area, the renosterbos (*D. rhinocerotis*), which is the shrub species defining this vegetation type. To extend the range of litter nutrient content, we also included the nutrient poor, common geophyte *Watsonia borbonica* (Pourret) Goldblatt. The nutrient element ratios of the selected litter types were as follows: (1) yellowbush (C:N = 23.0, and C:P = 367), (2) renosterbos (C:N = 52.4 and C:P = 810), and (3) *Watsonia borbonica* (hereafter *Watsonia*) (C:N = 133 and C:P = 8277). Owing to its nutrient-rich litter, the yellowbush enriches the soil under its canopy, producing fertile patches with higher levels of available nitrogen and phosphorus (Allsopp 1999; Simons and Allsopp 2007). *Watsonia* is a perennial geophyte which turns brown by the end of summer, and it is much more nutrient poor than the renosterbos.

Plant material was collected in early March 2007, at the end of the dry season. We cut the outer 5–10 cm branches of the year of healthy renosterbos and yellowbush shrubs, while whole senescent leaves of *Watsonia* were collected. The material was taken to the laboratory, dried at 40°C for at least 24 h and then stored in open containers at room temperature. We used leaves and thin branches (the latter from shrubs only) cut into approximately 1 cm long pieces. For each species, the air-dried litter was mixed thoroughly and then stored dry at room temperature until placed in the litter cages. For further details, see Bengtsson et al. (2011).

The air-dried litter was placed in cages consisting of cylindrical plastic containers (h = 4 cm, Ø = 7.5 cm), with a steel net bottom of 0.5 mm mesh size, and a removable lid with 1.6 mm mesh size. Each cage was filled with air-dried litter of one plant species up to approximately 3.5 cm and was weighed to nearest 0.1 mg (see Bengtsson et al. 2011). The cages were designed to give Collembola free access to the litter inside, and good drainage through the bottom. They were placed in the field on March 14, 2007, well before the onset of the wet season when the main decomposition and soil fauna activity was expected to occur, and sampled again for the extraction of animals from the litter at three occasions during the wet season; May 18, July 27, and September 12, 2007, that is, after 65, 131, and 182 days in the field (hereafter termed the first, second, and third sampling).

The cages allowed us to standardize a spatial configuration of litter type patches in an open system where the animals could freely move. Three sets of the three litter cage types were placed in level with the ground under five specimens of yellowbush and five of renosterbos at each of the two sites. The bushes were selected haphazardly in a way that ensured that both plant species were interspersed over the whole study site, to avoid confounding bush effects with unmeasured environmental gradients. The cages within each set were placed 3–4 cm from each other, while the distance between each set was at least 10 cm, all on the southern side of the bushes to minimize impact of sun exposure. At each sampling date, one randomly chosen set of cages was removed from each bush. After removal, each cage was placed in a plastic bag and transported to the laboratory in a coolbox. They were stored for no longer than 1 day at 10°C in a temperature controlled incubator, before being placed in a high gradient extractor (SMD Engineering, Stellenbosch University, South Africa) for 4 days (Leinaas 1978). After extraction, mass loss and chemical composition were analyzed for the litter from each cage separately (Bengtsson et al. 2011). Of the original 180 cages, 24 were accidentally lost to fire and 12 were lost for unidentified reasons. In addition to the originally balanced design, we also placed six cages of yellowbush litter (three under each bush species) from 27 July to 12 September. It appeared to decompose much faster than the other two litter types, and we wanted to determine the extent to which this nutrient-rich litter would attract Collembola at the end of the wet season when little was left of the original litter samples.

### Statistics

We analyzed the main data set with a General Linear Mixed Model using SAS procedure Mixed (SAS institute, Cary, NC). The fixed factors in the model were at level 1: Farm, Bush species, and their interaction Farm × Bush species, at level 2: Litter type and its interactions with the above factors, and at level 3: Sampling day and its interactions with all the factors above. The error terms were, when testing effects of factors at level 1: Farm × Bush species × Bush number (pair), at level 2: Litter × Farm × Bush species × Bush number, and at level 3 the residual error. The full model is given in Table 1 and was used for both dependent variables (abundance of *H. manubrialis*; summed abundances of all other Collembola species). Degrees of freedom were estimated with the Satterthwaite method. The mixed model takes the unbalanced design due to the loss of litter cages into account. The dependent variables were LN(N+0.1) (*H. manubrialis* abundance) or LN(N+1)-transformed before analysis.

**Table 1:** Results from a hierarchical mixed model (GLMM) analysis of the effects of bush species (G, R), farm (1, 2), litter type (G, R, W), and time of sampling (1, 2, 3) on (a) the number of *Hypogastrura manubrialis* (invasive) and (b) the sum of the numbers of all other Collembola species (indigenous). See text for further details on statistics. Factors with *P* < 0.01 are indicated in by bold

Effect	Num df	Den df	*F* Value	Pr > F
(a) *H. manubrialis* (LN N+0.1)-transformed				
Bush species	1	11.9	14.25	**0.0027**
Farm	1	11.9	2.35	0.151
Bush × Farm	1	11.9	1.96	0.187
Litter	2	26	34.58	**<0.0001**
Litter × Bush	2	26	1.69	0.204
Litter × Farm	2	26	1.86	0.176
Litter × Bush × Farm	2	26	1.32	0.286
Time	2	75.1	46.43	**<0.0001**
Bush × Time	2	75.1	6.82	**0.0019**
Time × Farm	2	75.1	3.04	0.0540
Bush × Time × Farm	2	75.1	3.05	0.0534
Litter × Time	4	75.2	7.04	**<0.0001**
Litter × Bush × Time	4	75.2	2.31	0.0654
Litter × Time × Farm	4	75.2	3.89	**0.0063**
Litter × Bush × Time × Farm	4	75.2	0.74	0.570
(b) Indigenous Collembola (LN (N+1)-transformed)				
Bush species	1	14.4	0.19	0.666
Farm	1	14.4	0.20	0.662
Bush × Farm	1	14.4	0.73	0.406
Litter	2	28.2	9.46	**0.0007**
Litter × Bush	2	28.2	0.93	0.408
Litter × Farm	2	28.2	0.44	0.650
Litter × Bush × Farm	2	28.2	1.31	0.285
Time	2	75.6	8.40	**0.0005**
Bush × Time	2	75.6	0.41	0.662
Time × Farm	2	75.6	10.28	**0.0001**
Bush × Time × Farm	2	75.6	0.27	0.767
Litter × Time	4	78.2	1.86	0.126
Litter × Bush × Time	4	78.2	0.21	0.932
Litter × Time × Farm	**4**	**78.2**	**4.37**	**0.0030**
Litter × Bush × Time × Farm	4	78.2	1.22	0.308

Differences between *H. manubrialis* and the indigenous species combined in their response to litter decomposition rate (k-values in individual litter cages; Bengtsson et al. 2011) were examined with ANCOVA, using LN(N+1)-transformed abundance data as the factor and k-value as covariate (JMP v. 8, SAS Institute). This general linear model was selected because it is reasonable to assume that litter decomposition rate in this experiment determines springtail abundance, a case when ordinary least square methods are more appropriate (Smith 2009), and because it gave a much better fit of the data compared to GLM models with Poisson errors, as indicated by residual plots. For simple comparisons, we also used nonoverlapping 95% confidence intervals as an indication of significant differences at the *P* < 0.05 level.

Correlations between *H. manubrialis* abundance and the total abundance and taxonomic richness (using operationally defined taxa; Table S1) of the indigenous species at each sampling date were examined by Spearman rank correlations, because the distributions of all variables were highly skewed toward low values (JMP 8). Differences in total abundance and taxonomic richness of indigenous species across all samples with more than 100 versus <100 *H. manubrialis* per litter cage were examined by One-way ANOVAs using LN-transformed dependent variables to obtain normality.

Differences in springtail community composition between litter types were examined by analysis of similarity (ANOSIM) followed by Multidimensional scaling (MDS), using Primer (PRIMER v 5.0, see Clarke and Warwick 2001). We used the Bray–Curtis similarity index on square-root-transformed abundances of the operationally defined taxa, but excluding taxa with mean abundance <1 individual/L cage. Because the Primer package cannot analyze the full hierarchical design of the field study, the analyses were carried out for each sampling date, first examining differences between farms, and then separately for each farm, with litter type nested under bush species. To determine effects of *H. manubrialis* on the species assemblage, the analyses were carried out with and without *H. manubrialis*. The ANOSIM procedure of Primer is a nonparametric permutation procedure applied to rank similarity matrices underlying sample ordinations, generating a global R-statistic ranging from 0 to 1, with higher R-values indicating more distinct assemblages (e.g., Clarke and Warwick 2001).

## Results

**Figure 1:**
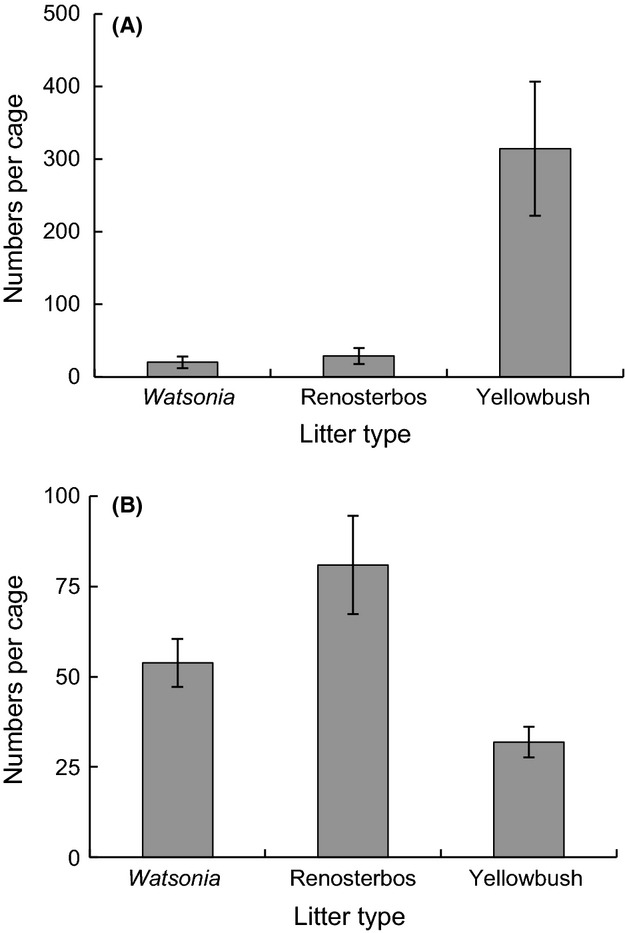
Numbers of individuals (±SE) per trap of each litter type, averaged over the three sampling dates. A = *Hypogastrura manubrialis*, B = all indigenous species combined. (Note differences in scale). *n*-values: 49 (Watsonia), 47 (renosterbos), 49 (yellowbush).

**Figure 2:**
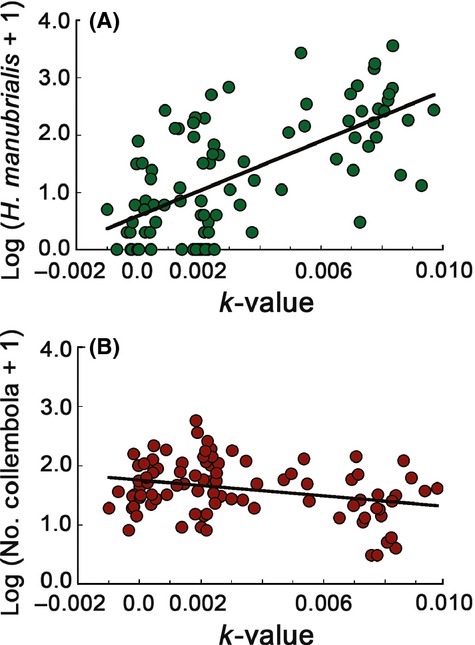
Numbers of animals per litter cage (LN (x +1)-transformed) as a function of litter decomposition rate in the same cage (abundance [*k*-values from Bengtsson et al. 2011], at time = 1 and time = 2 combined). (a) = *Hypogastrura manubrialis* and (b) = all indigenous species combined. Regression lines: *H. manubrialis: y* = 218.02*x* + 0.5846; *R*^2^ = 0.4275; *P* < 0.0001. Indigenous species: *y* = −44.315*x* + 1.7484, *R*^2^ = 0.0879; *P* = 0.0041. The slopes of the two relations differ significantly (significant interaction between k-value and taxa in ANCOVA).

The invasive *H. manubrialis* constituted almost 70% of all animals found in the study (Fig. 1). It was about an order of magnitude more numerous in the yellowbush than in the other litter types (Fig. 1, Table 1a). By contrast, the indigenous species showed the opposite trend, with the lowest abundance in the yellowbush litter (Fig1, Table 1b). The abundance of *H. manubrialis* varied greatly even within treatments. At the time of highest abundance (second sampling), the number of animals per yellowbush litter cage ranged from 0 to 3600 individuals. Across litter types and sampling dates, there was a positive relationship between litter decomposition rate and the number of *H. manubrialis* in individual litter cages (Fig2A). Again, all other Collembola species combined showed the opposite pattern (Fig2B). The difference in slope was significant, as indicated by the significant interaction term in the analysis of covariance (Taxa [*H. manubrialis* vs. all other]: *F* = 56.6, *P* < 0.0001; *k*-value: *F* = 21.2, *P* < 0.0001; Taxa × k-value: *F* = 51.4, *P* < 0.0001).

**Figure 3:**
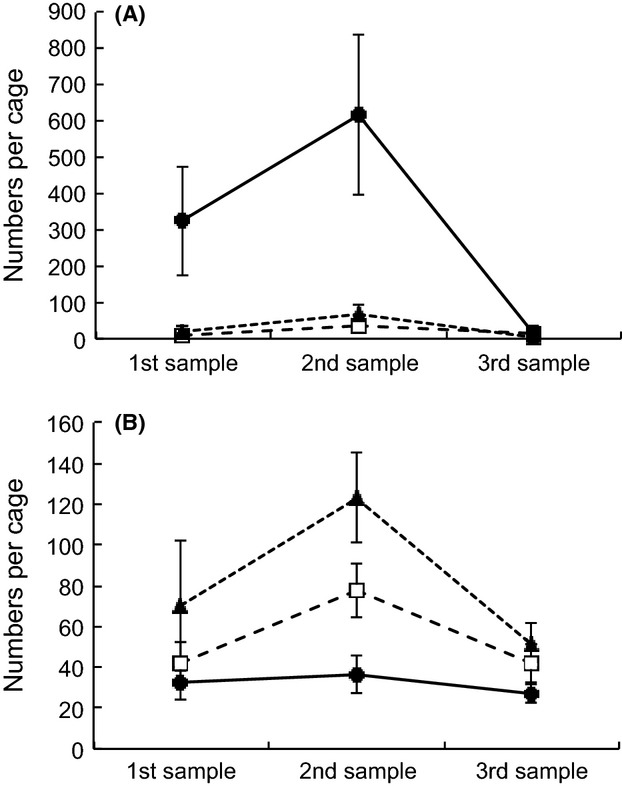
Mean numbers of animals per trap of each litter type on succeeding sampling dates. A = *Hypogastrura manubrialis*; B = All indigenous species combined. Black dots = yellowbush, Triangles = renosterbos, Squares = *Watsonia* (Note differences in scale).

Time and the litter × time interaction had significant effects on *H. manubrialis* abundance within cages (Table 1a). Peak abundance was found at the second sampling (Fig3), with the lowest abundance on the last sampling date. The decrease was most striking in the yellowbush litter, which by that time was strongly decomposed. A significant effect of bush species on *H. manubrialis* (Table 1a) showed that each litter type had the highest abundance of this species under yellowbush. This is illustrated in Figure S1. Most striking was the large difference in *H. manubrialis* occurrence between yellowbush litter under the two bush types. The final sampling also included six additional cages of yellowbush litter that had been placed out on the second sampling date. These samples were significantly less decomposed than the main trap series of yellowbush litter collected the same day (mean remaining proportion of original organic matter [±95% C.I.]: 0.44 [±0.051] vs. 0.32 [±0.020]; non-overlapping 95% C.I), and they hosted much higher numbers of *H. manubrialis* (244.5 [±130.6] vs. 16.0 [±75.4]; *n* = 6 and 18, respectively; nonoverlapping 95% C.I).

Significant effects of litter type and time on total abundance of the indigenous species were also found. As with *H. manubrialis*, the indigenous species had the highest abundance on the second sampling date, but they differed from the former species in litter type occupancy, being least abundant in yellowbush litter. The differences between litter types persisted throughout the experimental period; that is, there was no significant litter × time interaction (Fig. 3, Table 1b). Moreover, bush species had no significant effect on the abundance of the indigenous springtail species.

**Figure 4:**
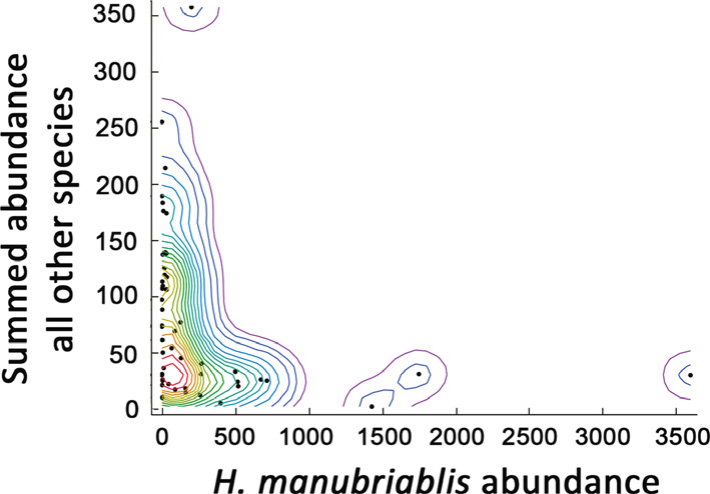
Relationship between the abundance of *H. manubrialis* and of all other species combined in each litter cage at the second sampling date. The lines indicate 5% quantiles using the Nonparametric Bivariate Density plot function (JMP 8 for Macintosh; SAS Institute). The relationship is significantly negative (Spearman rank correlation, *r*_*s*_ = 0.40, *P* = 0.0037).

The summed abundance of the indigenous species was negatively correlated with the abundance of *H. manubrialis* on the second sampling date (Spearman rank correlation, *r*_*s*_ = 0.40, *P* = 0.0037, *n* = 51) (Fig. 4), but not at the first and third sampling dates. No significant relationships between the abundance of *H. manubrialis* and taxonomic richness of indigenous species occurred at any sampling date. When combining the data from all dates and litter types, cages with <100 *H. manubrialis* had higher abundance of indigenous species than those with more than 100 *H. manubrialis* (ANOVA: mean (±SE) in samples with less vs. more than 100 *H. manubrialis*: 60.9 (±6.46) vs. 42.7 (±12.9), *F* = 4.09, *P* = 0.046; *n* = 116 and 29, respectively). Moreover, at the second sampling, significant effects of high *H. manubrialis* abundance (>100 inds.) were found for both abundance and species richness of indigenous species (ANOVAs: Abundance: *F* = 15.73, *P* = 0.0002; Richness: *F* = 4.21, *P* = 0.046; *n* = 34 (low) and *n* = 17 (high)). Thus, the indigenous species and *H. manubrialis* responded in opposite ways to the experimental conditions both between and within litter types.

At the two-first sampling dates, but not the last one, ANOSIM with *H. manubrialis* included showed significant differences in community composition between litter types for both farms (Global R: Farm 1, 0.398 at *t* = 1 (*P* = 0.001), 0.16 at *t* = 2 (*P* = 0.021). Farm 2, 0.525 (*P* = 0.004) at *t* = 1, 0.380 (*P* = 0.006) at *t* = 2), but not between bush species (no Global R-values were significant). The differences between litter types were largely the consequence of abundance variation of *H. manubrialis*. When only the indigenous species were included in the analyses, a significant difference was only found at one farm on the first sampling date (Global R: Farm 1, 0.232 at *t* = 1 (*P* = 0.008), 0.047 at *t* = 2 (ns). Farm 2, 0.106 (ns) at *t* = 1, 0.089 (ns) at *t* = 2). This limited effect of litter type on community structure when only including indigenous species means that although densities of these species varied greatly between litter types, their relative numerical composition did not. Thus, a negative correlation with *H. manubrialis* abundance, notably in the yellowbush litter, appears to reflect a fairly uniform response pattern among the indigenous species.

## Discussion

Habitat disturbance may have considerable impacts on species invasions and lead to complex interactions between direct and indirect effects of invasion (Kocher and Williams 2000; Hansen and Clevenger 2005; MacDougall and Turkington 2005; Alston and Richardson 2006; Didham et al. 2007). In our study, the occurrence of *H. manubrialis* was clearly dependent on the nutrient-rich yellowbush litter. The rarity of the species elsewhere in the region is further evidence of this effect (Janion 2012; Liu et al. 2012; Janion-Scheepers et al. 2015). Supply of this litter type to the renosterveld system is promoted by overgrazing (Allsopp 1999). Consequently, the abundance of *H. manubrialis* invasion appeared dependent on both processes. Our study emphasizes the potential importance of positive species interactions in determining over-all effects of environmental change on species invasion (see Simberloff and Von Holle 1999; Bruno et al. 2003). The yellowbush produces nutrient-rich litter that decomposes much more quickly than that of renosterbos and *Watsonia* (Bengtsson et al. 2011) and thus may create patches of rich but ephemeral resources to Collembola. The invasive *H. manubrialis* appeared to be very efficient in utilizing these high quality patches. In fact, the interaction with the yellowbush appears so important in determining the spatial distribution of the species that it is questionable whether the species had been able to invade the area if the yellowbush had not been present. This is consistent with the species typically being associated with rich soils (Fjellberg 1998), and rare or absent from much of the region in undisturbed habitats. Here, its sparse occurrence in the other litter types may well depend on dispersal from the rich patches of yellowbush litter, in a source-sink dynamics (e.g., Pulliam 1988).

Previous reports of *H. manubrialis* from South Africa involve scattered observations from urban areas or cultivated habitats (Womersley 1934; Paclt 1967), but not from the rather nutrient poor fynbos vegetation (Janion et al. 2011; Janion 2012; Liu et al. 2012; Janion-Scheepers et al. 2015). In our study, the dense aggregations of up to several thousand animals within individual litter cages show that yellowbush litter represents a highly favorable food resource for *H. manubrialis*. This is also indicated by the effect of bush identity; the highest abundance of *H. manubrialis* within each litter type was found under yellowbush, with most striking effect with the yellowbush litter. However, dense aggregates (>500 inds.) were only found in a fraction (7 of 16) of the yellowbush litter cages even at the time of the second sampling, suggesting that no litter cage could support the extremely high density of *H. manubrialis* for long periods of time.

The results also suggest that the characteristics of individual species may be significant for the success and impact of species invasion under a given situation. The efficient utilization of temporary high quality patches by *H. manubrialis* seems to be a consequence of its high mobility and ability to coordinate group migration (Simon 1975). Similar group behavior has been described in several closely related species (Lyford 1975; Mertens and Bourgoignie 1977; Leinaas 1983). The distribution of *H. manubrialis* at the third sampling, being abundant only in the six additional cages with less decomposed litter, is consistent with group behavior enabling the species to aggregate in favorable patches and leave when resources are exploited. Other invasive collembolan species reported from Western Cape (Janion et al. 2011) were not found in the present study area. None of them have similar group behavior as *H. manubrialis*.

**Figure 5:**
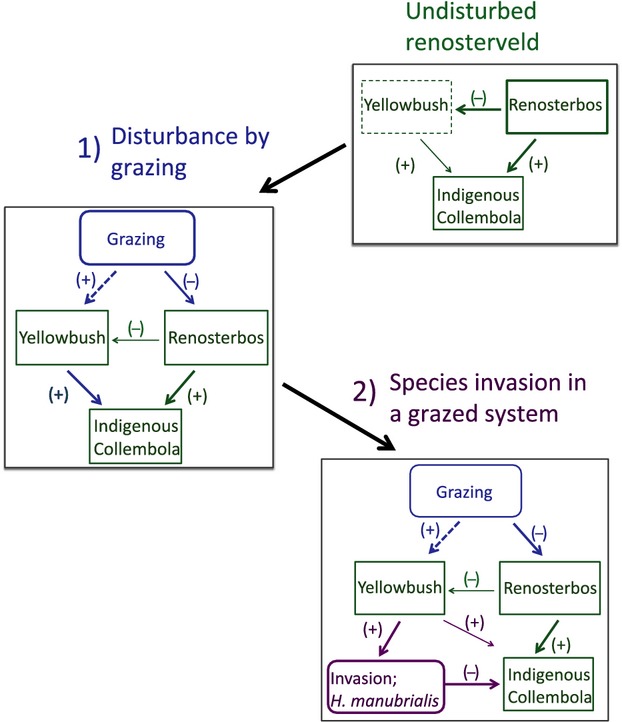
Hypothetical framework for the effects of grazing and invasion of Hypogastrura manubrialis on indigenous Collembola: Native renosterveld (green); when undisturbed this vegetation type is dominated by renosterbos. The competitively inferior yellowbush is uncommon (dotted rectangle). (1) Livestock grazing (blue) has negative effects on the renosterbos, but indirectly favours (dotted arrow) the grazing resistant yellowbush. It produces nutrient rich litter that likely improves resources for the indigenous Collembola. (2) Invasion of the alien Collembola H. manubrialis (purple) is facilitated by increased abundance of yellowbush, likely resulting in a negative effect on the indigenous fauna in patches dominated by this rich litter. Arrow coloration refers to changes in interactions from one scenario to the next. Thick arrows = hypothesized major or strongly increased impact of interactions. Thin arrows = hypothesized less important or strongly reduced impacts.

Although species interactions were not experimentally tested is this study, correlation analyses suggested a significant effect of *H. manubrialis* on the other Collembola. In general, one would expect that the highest collembolan density and species diversity would be found in the patches of highest quality (e.g., Hertzberg et al. 2000; Salamon et al. 2004; Terauds et al. 2011). However, in this study, the indigenous species had the lowest abundance in the nutrient-rich yellowbush litter, and there was a negative correlation between the decomposition rate and number of animals per sample. The similarity in responses between the indigenous species in this respect agrees with the fact that all taxa, with sufficient abundance to make statistical analyses meaningful, had lowest abundances in the yellowbush litter (i.e., *Brachystomella* sp.; *Xenylla* sp.; *Parisotoma* sp.; *Lepidocyrtus* sp. and Symphypleona; H.P.Leinaas et al., unpublished results). It seems unlikely that the indigenous species actually prefer more nutrient poor litter, as all litter types are native and thus familiar to them. Although examination of species interactions require a different experimental design, it is difficult to explain the opposite responses of the indigenous species other than that they most likely reflect a negative interaction with *H. manubrialis,* which had high abundance only in the yellowbush litter cages. This idea is further supported by the negative correlation between the densities of *H. manubrialis* and indigenous species at the second sampling date, when the former species showed peak abundance (Fig. 4), and by the fact that samples with many *H. manubrialis* had lower abundance and species diversity of indigenous species than samples with few *H. manubrialis*. Moreover, a similar study made in 2008 in a fynbos site where *H. manubrialis* has not been observed, showed more indigenous Collembola in yellowbush litter than in the three other litter types used (Janion 2012). In fact, in that investigation, the yellowbush litter had higher abundances of indigenous species than observed in the identically treated cages of the present study (September sampling in both studies, means (±95% C.I.): 66.4 (±28.7; *n* = 27) in fynbos vegetation vs. 27.1 (±9.27; *n* = 18) in the present study in renosterveld). Thus, in the absence of *H. manubrialis*, yellowbush litter appears favorable to the indigenous species. The outcome appears to be a an interaction between disturbance, colonization by yellowbush, the ability of *H. manubrialis* to rapidly reach high abundances on the litter of this plant, and a negative effect of this species on other springtail species. This proposed interaction hypothesis is illustrated in Figure5 to enable the further field experimental work that will be required to test it fully.

Habitat heterogeneity has been recognized as an important factor affecting both invasion and impacts on native species assemblages, although interpretations may vary, partly due to differences in scale (Marvier et al. 2004; Davies et al. 2005; Fridley et al. 2007; Melbourne et al. 2007). On the small scale, some have argued that habitats suitable for indigenous species are likely also to be suitable for introduced species, and invasion thereby leading to the most species-rich patches becoming even more species rich, while others emphasize that indigenous diversity may improve resistance against invasion in species-rich habitats (see e.g., Stohlgren et al. 1999; Fargione and Tilman 2005). Our results suggest a contrasting scenario that an invader may be so strongly favored by suitable conditions that it drastically reduces indigenous species abundances. On the other hand, the fact that *H. manubrialis* seems able to utilize lower quality litter to a much more limited extent suggests that low quality litter in this area may provide the indigenous species with a spatial refuge., Thus, habitat heterogeneity may be playing a role in maintaining the overall species diversity in the area (Melbourne et al. 2007).

In conclusion, our work provides an illustration of how the co-occurrence of species invasion with other types of ecological change can represent an important challenge for understanding the mechanisms underlying and the likely outcomes of environmental change. Understanding the likelihood of such “ecological surprises”, and exploring their likely mechanistic basis, remain important areas in ecology (Paine et al. 1998).

## Data Accessibility

The data will be archived in Dryad.

## Supporting Information

**Figure S1.** Abundances of *H. manubrialis* and indigenous Collembola in different litters under different bush species. **Table S1.** Collembola taxa distinguished in the study.
