# Supplementary material for: Indirect effects of habitat disturbance on invasion: nutritious litter from a grazing resistant plant favors alien over native Collembola
Source: Ecol Evol. 2015 Jul 25;5(16):3462–71. doi: 10.1002/ece3.1483 (PMC4569040; doi:10.1002/ece3.1483)

**Supporting Information Table S1**

Collembola taxa (genera in italics) distinguished in the study. Systematics according to Checklist of the Collembola (<http://www.collembola.org/taxa/collembo.htm> - Last updated on 2012.01.31). Taxa in which we have observed more than one morpho-species are denoted with “spp.” All non-invasive taxa are classified as indigenous, although there are some uncertainties about the status of *Entomobrya* in South Africa (only 0.7 % of the material).

**Indigenous:**

Hypogastruridae

*Ceratophysella*

*Xenylla*

Brachystomellidae

*Brachystomella*

Pseudochorutinae

Pseudochorutinae

Neanurinae

Neanurinae

Onychiuridae

Onychiuridae

Isotomidae

*Parisotoma* spp*.*

*Cryptopygus*

Other Isotomidae

Entomobryidae

*Entomobrya* spp

*Lepidocyrtus* spp*.*

*Seira*

Symphypleona

Symphypleona spp.

Other unknown springtail

**Invasive:**

*Hypogastrura manubrialis*

**Supporting Information Figure S1**

Abundances of (A) *H. manubrialis* and (B) all indigenous species combined in different litters (yellowbush, renosterbos, *Watsonia*) placed under different bush species (yellowbush Y, or renosterbos R). Non-transformed LS means (±Standard Error) are shown; n-values vary between 23 and 26. *Note* differences in y-axis scale.

The figure shows the non-transformed data from the file submitted to Dryad, and does not represent the model presented in Table 1a-b, in which the dependent variables were LN-transformed and additional variables (farm, bush number, time) representing the hierarchical structure of the study were included. *Note* that the Litter*Bush interaction was not significant for any of the two dependent variables when these were transformed to obtain normality (Table 1). See main text for further explanations.


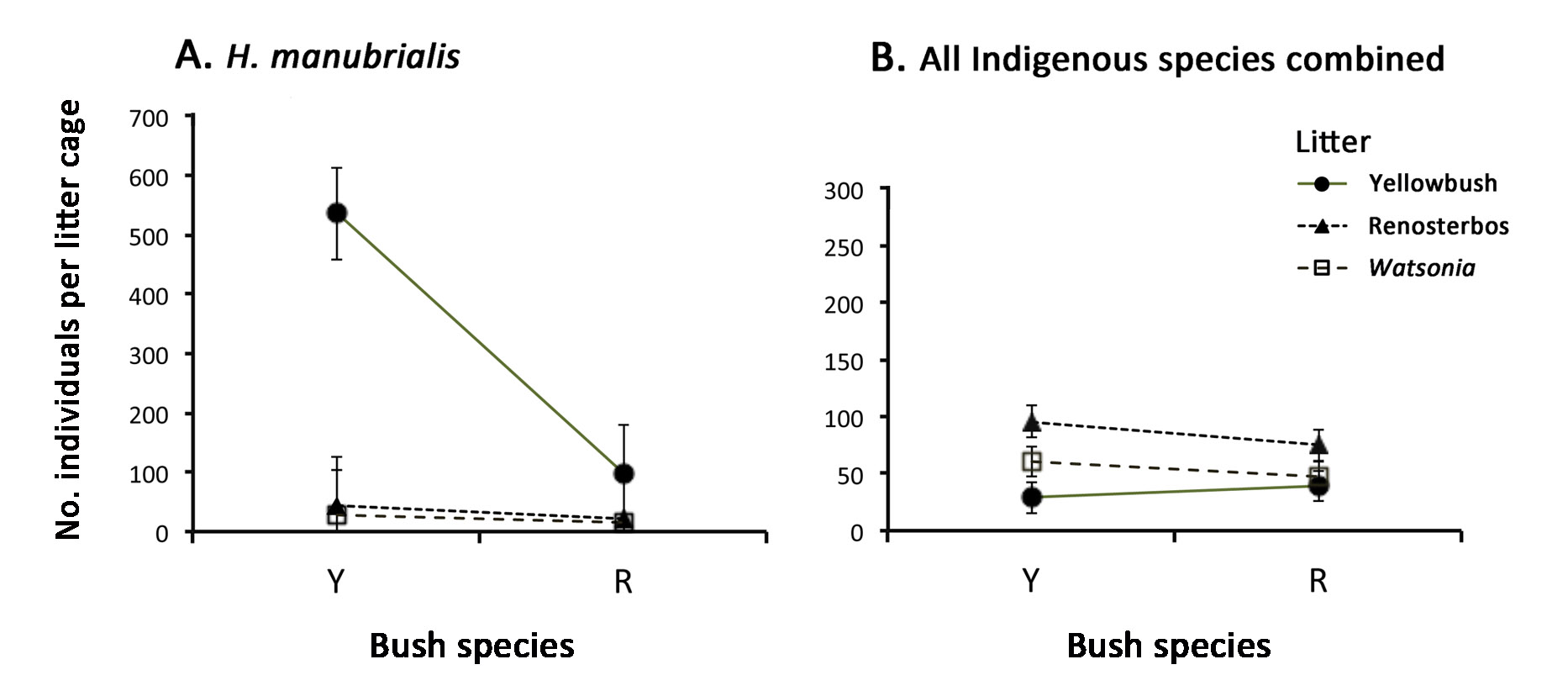

Supplement: Supplementary file 1 [file ece30005-3462-sd1.docx]
